# Analysis of Microbial Interactions During the Production of Chinese Ethnic Fermented Foods

**DOI:** 10.3390/foods15030489

**Published:** 2026-02-01

**Authors:** Xinyue Jiang, Xianghao Li, Panpan Song, Yao Dou, Jiayi Xue, Ze Wu, Shuaijun Ma, Wuxuan Wei, Wenjing Zheng, Shaohua Dou, Liang Dong

**Affiliations:** 1 College of Life and Health, Dalian University, Dalian 116622, China; 13199062388@163.com (X.J.); hao_her@139.com (X.L.); 18045915251@163.com (P.S.); 17741095029@163.com (J.X.); williamwu2025@163.com (Z.W.); ms767929410j@163.com (S.M.); 18042621620@163.com (W.W.); 17667376687@163.com (W.Z.); 2 College of Economics and Management, Huazhong Agricultural University, Wuhan 430070, China; angelyao22@163.com

**Keywords:** microbial interactions, fermented foods, ethnic minorities, flavor formation, microbiome regulation, modernization of traditional foods

## Abstract

Food fermentation is an ancient bioprocess characterized by complex biochemical transformations driven primarily by microbial communities. Across the diverse regions of China, various ethnic groups have developed a rich array of traditional fermented foods through long-term practical experience. These foods are integral to local culinary heritage and provide valuable systems for studying microbial ecology and function. From the perspective of microbial interactions, this review summarizes key concepts and major interaction types—including mutualism, commensalism, and competition—and describes how bacteria, yeasts, and molds interact via metabolic division of labor to drive substrate conversion, flavor formation, preservation, and biosynthesis of functional compounds. Focusing on four representative ethnic fermented foods—Dong fermented fish, Mongoslian milk curd, Miao sour soup, and Manchurian kombucha—we analyze how microbial interactions contribute to product quality, safety, and sensory attributes. Given current challenges in industrializing traditional fermented foods, such as poor standardization and variable quality, we propose future research directions centered on modern microbiome tools, designed microbial consortia, and process optimization. This work aims to provide a scientific foundation and practical strategies for modernization and quality improvement of traditional fermented foods.

## 1. Introduction

**Figure 1:**
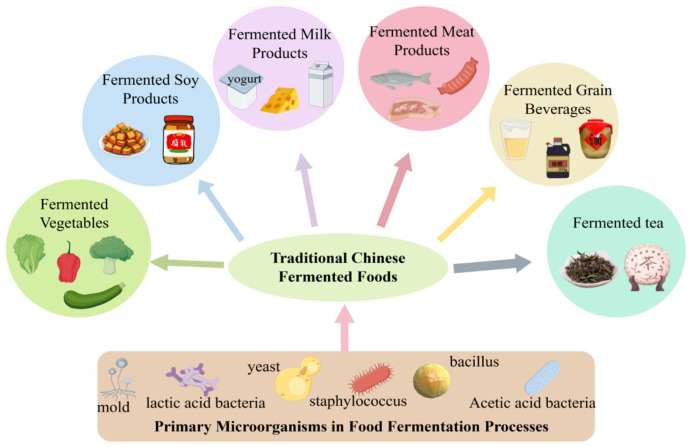
Traditional Chinese Fermented Foods.

As a multi-ethnic nation, China has developed a rich and diverse culinary culture through the long-term historical practices of its fifty-six ethnic groups. Among these traditions, fermented foods—whether produced by natural fermentation or through inoculation with traditional starters—occupy an essential role in the daily diets and ceremonial practices of various communities. Their significance stems from sensory qualities [1], nutritional value [2,3], and reported health benefits [4,5,6,7,8,9]. Figure 1 presents an overview of traditional Chinese fermented foods, spanning categories such as fermented vegetables, soy products, dairy, meat, grains, and tea. Representative examples include sour meat [10] and sour fish [11] from southwestern regions, cheeses [12] and yogurts [13] associated with northern pastoral groups, and sour soups [14] and fermented soybean products [15] prevalent across various localities. These foods are not merely products of adaptive ingenuity in response to local environmental conditions; they also constitute a form of “living heritage,” deeply embedded with ethnic identity and cultural memory. Selected traditional fermented foods emblematic of China’s culinary heritage are further summarized in Table 1.

Food fermentation involves the dynamic succession of microbial communities that convert substrates into biomass and diverse metabolites [16,17]. Within these systems, microorganisms engage in ecological interactions—such as mutualism, commensalism, and competition—which collectively enhance food safety, nutritional quality, and flavor development [18,19]. Key mechanisms include the production of antimicrobial compounds, nutrient competition and cross-feeding, ion acquisition, signaling, pH modulation, and biofilm formation [20]. In this context, “microbial interactions” refer to relationships that lead to measurable functional outcomes—such as flavor formation or safety enhancement—through mechanisms like cross-feeding or environmental modification. Correlations derived solely from omics-based analyses are insufficient to establish causality.

While pure-culture fermentation is well-established industrially, many traditional ethnic fermented foods derive their distinctive sensory and functional qualities from complex, naturally formed microbial consortia. The flavor complexity and functional diversity achieved through such interactive fermentation are difficult to replicate using single strains. Therefore, elucidating the mechanisms behind microbial interactions in traditional foods not only provides a scientific reappraisal of ethnic food heritage but also lays a theoretical foundation for developing novel starter cultures [21], optimizing fermentation processes [22,23], and advancing the standardization and industrialization of traditional production [24,25,26]. Such research is vital for preserving biological and cultural diversity and supporting sustainable local economies.

This review summarizes fundamental principles of microbial interactions and then examines their roles in representative fermented foods from China’s ethnic minorities. By synthesizing evidence on how complex microbial communities transform raw materials via ecological and metabolic networks, we further discuss how modern scientific approaches can be used to characterize, preserve, and improve these traditional bioprocesses.

**Table 1:** Fermented Foods with Chinese Characteristics.

Types of Fermented Foods with Chinese Characteristics		The Microbial Strains Used in the Fermentation Process	References
Fermented soybean products	Soy sauce	*Aspergillus oryzae*, *Aspergillus sojae*, *Aspergillus niger*	[27]
	Fermented bean curd	*Enterococcus*, *Lactococcus*, *Geotrichum*, *Mortierella*, *Bacillus cereus*	[28]
	Soybean Paste	*Aspergillus oryzae*, *Biscudii yeast*, *Bacillus*, and *Lactobacillus plantarum*	[29,30]
Fermented grain products	Vinegar	*Lactobacillus*, *Lacticaseibacillus*, *Lentilactobacillus*, *Limosilactobacillus*, *Leuconostoc*, and *Pediococcus.*	[31]
	Baijiu	*Lactobacillus*, *Thermoactinomyces*, *Aquabacterium*, *Aspergillus*, and *Kazachstania*	[32]
	Rice wine	*Lactobacillus plantarum*, *Lactobacillus paracasei*, *Leuconostoc mesenteroides* and *Saccharomyces cerevisiae*	[33]
Fermented Vegetable Products	Pickled cabbage	*Latilactobacillus sakei*, *Loigolactobacillus coryniformis* subsp. *torquens*, *Lactiplantibacillus plantarum* subsp. *plantarum*, and *Secundilactobacillus malefermentans*	[34]
	Kimchi	*Lactococcus*, *Weismannia*, and *Lactobacillus*	[35]
Fermented meat products	Pickled Fish	*Lactobacillus plantarum*, *Streptococcus*, *Staphylococcus*, *Bacillus*, *Micrococcus*, *Pseudomonas*, *Candida*, *Saccharomyces cerevisiae*, and *Saccharomyces*	[36]
	Ham	*P. urinaeequi*, *P. pentosaceus*, and *L. pentosus*.; *S. xylosus*, *S. equus*, *S. gallinarum*	[37]
Fermented dairy products	Cheese	*Lactococcus*, *Streptococcus*, *Lactobacillus*, *Leuconostoc*, and *Enterococcus*	[38]
	Yogurt	*Lactobacillus*, *Streptococcus*, *Streptococcus*, *Leuconostoc*, *Bifidobacterium*, and *Peptostreptococcus*	[39]

## 2. Microbial Interactions

**Figure 2:**
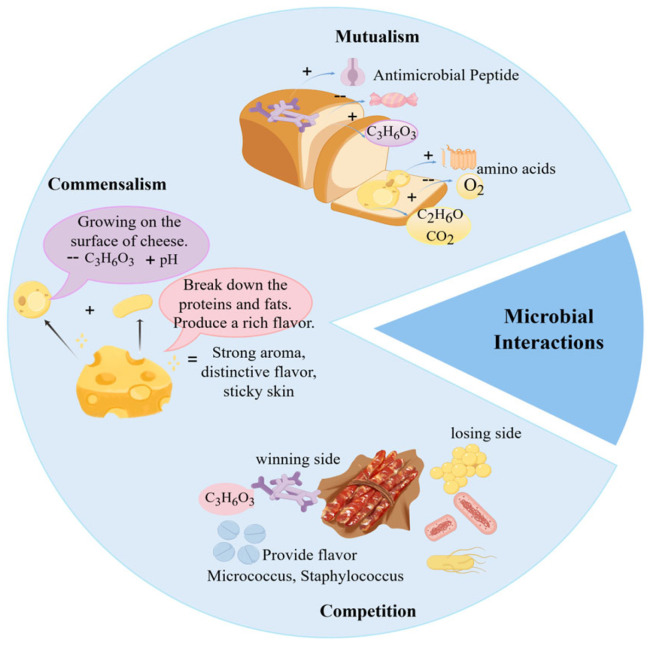
Common Microbial Interactions in Food Fermentation.

Microbial interactions play a pivotal role in food fermentation, not only determining fermentation success but also serving as the fundamental mechanism underlying the distinct sensory properties and safety of fermented products. Through cooperative and competitive relationships, acid-producing bacteria, oxygen-consuming microorganisms, and producers of antimicrobial substances collectively modify the fermentation milieu, selectively favoring the proliferation of beneficial microbes and thereby substantially improving product biosafety [40]. In contrast to the often monotonous flavor profiles resulting from single-strain fermentation, synergistic microbial activities give rise to complex, well-balanced, and multi-layered flavor systems [3,41,42]. Moreover, these interactions directly influence the physical structure of food, functioning both as a natural preservation strategy and as a key contributor to the enhancement of overall product quality and value [43,44,45]. As shown in Figure 2, common microbial interactions in food fermentation include mutualism, commensalism, and competition.

### 2.1. Mutualism

Mutualism describes a close and interdependent interaction between two or more microorganisms, in which all participating members derive benefit from the relationship. It is characterized by coexistence and reciprocal dependence. In nature, over 98% of microorganisms exhibit some form of nutritional auxotrophy, lacking key genes or pathways necessary for the synthesis of essential metabolites [46]. These microorganisms rely on acquiring such nutrients from their environment to maintain vital cellular functions, making mutualistic relationships a widespread phenomenon in microbial ecosystems [47]. In food fermentation, whether through direct inoculation of specific microbes or by selectively promoting the growth of indigenous microbial populations, mutualistic interactions contribute to the production of organic acids, ethanol, and other metabolic end-products. These metabolites, in turn, enhance food safety, extend shelf life, and improve the sensory and functional qualities of fermented foods [48]. However, the extent of mutualistic benefits is often context-dependent, varying with strain specificity, substrate composition, and processing conditions.

Multiple studies indicate that interactions among diverse microbial communities can generate unique sensory characteristics. For example, in coffee fermentation, yeasts (such as *Pichia kudriavzevii*, *Torulaspora delbrueckii*, *Hanseniaspora uvarum*, *Candida railenensis*, *Wickerhamomyces anomalus*, etc.) and bacteria (e.g., *Leuconostoc mesenteroides*, *Lactiplantibacillus plantarum*) are commonly involved [49,50,51]. The application of defined microbial starters has proven effective in modulating flavor quality. Sequential inoculation with *Lactobacillus plantarum* and *Saccharomyces cerevisiae*, for instance, enhances fruity and fermented notes while increasing the diversity of volatile compounds in coffee [52]. Similarly, coffee co-inoculated with *Saccharomyces cerevisiae* and *Bacillus amyloliquefaciens* achieves higher sensory evaluation scores [53]. In Swiss cheese fermentation, Ilhan [54] observed that *Lactococcus lactis* supplies lactic acid as a substrate and establishes a favorable environment for *Propionibacterium*, while *Propionibacterium* in turn creates structural pores that facilitate the growth of *Lactococcus lactis* and contributes to characteristic flavor development. Furthermore, Tong et al. [55] demonstrated that varying the inoculation ratio of *Lactobacillus bulgaricus* and *Streptococcus thermophilus* significantly influences the fermentation kinetics and final properties of yogurt. These strains exhibit cross-feeding through the exchange of growth factors such as amino acids and formic acid, working collectively to accelerate acidification and aroma formation.

### 2.2. Commensalism

In fermentation systems, commensalistic interactions among microorganisms contribute to the development of complex flavor profiles in fermented foods. Commensalism describes a relationship in which one microorganism benefits from another without harming or benefiting the latter [56]. In practice, commensalistic effects may be difficult to distinguish from succession or indirect interactions without targeted validation. Such interactions can shape novel flavors by supporting the growth or activity of certain fermentative microbes, without negatively affecting the dominant fermenting populations. For instance, a Japanese research team reported that the characteristic flavor of white mold cheese arises from aromatic compounds generated through intricate bacterial–fungal interactions. Lactic acid bacteria lower the internal pH of the cheese, creating a favorable environment for the surface growth of the white mold *Penicillium*, which in turn forms the distinctive rind. The study further confirmed that volatile metabolites produced during this process significantly contribute to the unique aroma of the cheese [57]. Similarly, in soy sauce fermentation, Liu et al. [27] highlighted the roles of the koji fermentation and mash fermentation stages. During koji fermentation, molds such as *Aspergillus oryzae*, *Aspergillus niger*, and *Aspergillus flavus* enzymatically hydrolyze proteins and starches into short-chain compounds. These breakdown products then serve as substrates for lactic acid bacteria and yeasts during mash fermentation, where they are further converted into organic acids and aroma compounds that define the flavor of soy sauce. Additionally, Canon et al. [58] observed that proteolytic lactic acid bacteria can supply branched-chain amino acids to non-proteolytic strains, thereby promoting the growth of the latter without compromising their own metabolic activity. This finding offers a theoretical basis for designing fermented foods with improved functional properties or for developing novel fermentation substrates.

### 2.3. Competition

Competitive interactions occur when two or more microorganisms inhibit each other’s growth by competing for limited resources [59]. While some microbes may temporarily suppress others through the secretion of inhibitory metabolites, such effects are often transient. Over time, resistant subpopulations can emerge among the inhibited strains, subsequently competing with the dominant microbes for nutrients and ecological niches, thereby challenging their supremacy [60].

A representative example can be observed during kimchi fermentation. Initial colonizers such as *Enterococcus faecalis* rapidly utilize simple sugars (e.g., glucose and fructose) to produce acid and carbon dioxide, creating an acidic environment that inhibits competitors like *Escherichia coli*. As the pH drops and readily available sugars become depleted, the competitive fitness of *E. faecalis* declines. More acid-tolerant species, such as *Lactobacillus plantarum*, which can metabolize a broader range of carbohydrates, then gain a competitive advantage and become dominant in the mid-fermentation stage, driving further acidification. In the late stage, extremely low pH and nutrient scarcity suppress nearly all bacterial activity. At this point, acid-tolerant yeasts capable of utilizing residual complex carbon sources gradually become more competitive and initiate slow growth. This dynamic illustrates how shifts in environmental conditions continuously reshape the competitive landscape, leading to the succession of dominant microbial communities throughout fermentation [61,62,63].

Competitive mechanisms critically shape winemaking. *Saccharomyces cerevisiae* rapidly converts sugars into ethanol—a compound broadly inhibitory to many microorganisms. Its early metabolic activity also lowers pH, further suppressing competitors. Some strains even secrete proteinaceous toxins targeting wild yeasts. By thriving anaerobically and consuming oxygen, *S. cerevisiae* additionally restricts aerobic competitors like acetic acid bacteria and molds, securing dominance [64,65,66].

As highlighted in a review by Torres [67], ethanol stands out as the most significant inhibitory compound in alcoholic fermentations, with *Saccharomyces cerevisiae* being the primary producer of ethanol at high concentrations. Ashaolu et al. [68]. noted that lactic acid bacteria–dominated fermentations not only improve nutritional and sensory attributes but also enhance food safety through the production of antimicrobial compounds such as carbon dioxide, short-chain organic acids, hydrogen peroxide, diacetyl, and lactoperoxidase systems. These metabolites can help reduce aflatoxin levels, detoxify harmful substances, suppress pathogenic contamination, and lower the energy required for cooking, thereby improving the overall safety profile of grain-based fermented foods. The influence of microbial competition and other ecological interactions on the properties of representative fermented foods is summarized in Table 2. Notably, inhibitory outcomes are often modulated by environmental factors (e.g., pH, oxygen availability, and nutrient limitation) and may vary across strains and processing regimes.

### 2.4. Shared Mechanisms Underlying Microbial Interactions in Food Fermentations

Fermentation outcomes are shaped by microbial interactions across diverse systems. Early community assembly is initiated by environmental remodeling: pioneer microorganisms acidify the matrix or consume oxygen, thereby enriching functional taxa and suppressing competitors. For example, oxygen depletion by *Aspergillus* can favor vitamin B12 production by propionic acid bacteria [69]; oxygen availability drives bacterial succession and flavor development in Huangjiu wheat Qu [70]; and acidification by acid-tolerant bacteria inhibits pathogens [71]. Community stability and metabolic efficiency are sustained by metabolic complementarity and cross-feeding. Typically, yeasts or molds supply vitamins and amino acids to auxotrophic lactic acid bacteria (LAB), whereas LAB produce organic acids that lower pH and favor acid-tolerant populations [72], while promoting efficient macromolecule conversion.

Representative interaction modules include: (1) LAB–yeast consortia in sourdough and fermented beverages, where yeasts provide growth factors and LAB release assimilable nitrogen via proteolysis [73,74,75]; (2) yeast–acetic acid bacteria (AAB) consortia in kombucha and vinegar, in which yeast-derived ethanol is oxidized by AAB to acetic acid, generating an acidic environment that limits spoilage organisms [76,77]; and (3) mold–bacteria partnerships in soy sauce and cheese, where fungal extracellular enzymes (e.g., from *Aspergillus* spp.) liberate oligosaccharides and peptides that support bacterial growth and flavor biosynthesis [78,79]. These interactions also determine sensory properties: downstream conversion of ethanol and organic acids into volatile compounds (e.g., esters) drives aroma and flavor complexity [80], whereas nutrient competition and antimicrobial metabolites (e.g., bacteriocins) further modulate community structure and succession [81,82]. Collectively, fermentation is governed by coupled processes of environmental selection, metabolic cooperation, and competition that underpin ecosystem robustness and determine final product quality and safety.

**Table 2:** Effects of Microbial Interactions on Fermented Foods.

Function Type	Fermented Foods	Primary Strains	Interactions Between Strains	References
Mutualism	Yogurt	*Streptococcus thermophilus* and *Lactobacillus delbrueckii* ssp. *bulgaricus*	*S. thermophilus* provides formic acid, folic acid, carbon dioxide, and fatty acids to initiate the growth of *L. bulgaricus*. *L. bulgaricus* produces excess peptides and free amino acids to meet the biosynthetic demands of *S. thermophilus*.	[83]
	Fermented bean curd	*Mucor*, *Lactobacillus*, *Bacillus* and *Saccharomyces*	*Mucor* decomposes proteins and starch into peptides, amino acids, and monosaccharides, which are then utilized by yeast to produce alcohol, esters, and other aromatic compounds.	[84]
	Sourdough Bread	*Saccharomyces exiguus* and *Lactic acid bacteria*	Lactic acid bacteria convert sugars in flour into lactic acid and acetic acid, which yeast utilizes. The carbon dioxide produced by yeast causes the dough to rise, creating more space for lactic acid bacteria to thrive.	[85]
Commensalism	Cheddar cheese	*Streptococcus thermophilus* and *Lactococcus strains*	*S. thermophilus* has a crucial role in boosting *Lactococcus* growth	[86]
	Soy sauce	*L. Fermentum* and *Zygosaccharomyces rouxii*	*L. Fermentum* alleviates the inhibitory effect of acetic acid on *Zygosaccharomyces rouxii*, and its metabolites promote the growth of *Zygosaccharomyces rouxii*	[87]
	Baijiu	*S. cerevisiae* and *Lactobacillus buchneri*	Yeast and lactic acid bacteria synergistically enhance product yield while mutually supplying nutrients to promote growth and metabolic activity.	[32]
Competition	Marula wine	*Lactobacillus*, *Lactobacillus plantarum*; *luconobacter oxydans*, *Acetobacter pasteuriannus*	Lactic acid bacteria inhibit the growth of acetic acid bacteria through malic acid-lactic acid fermentation.	[88]
	Sausage	*Debaryomyces hansenii (yeast)*, *Penicillium* and *Aspergillu* competing for limited resources	Yeast and mold protect the fermentation process internally by competing for resources, contributing unique flavors and textures to the sausage casing. Bifidobacteria metabolize carbohydrates to produce lactic acid and small amounts of acetic acid, lowering the product’s pH.	[89]

## 3. Microbial Interactions in Fermented Foods of China’s Ethnic Minorities

### 3.1. Dong Ethnic Fermented Fish: A “Sour-Savory Symphony” Driven by Lactic Acid Bacteria and Yeast

**Figure 3:**
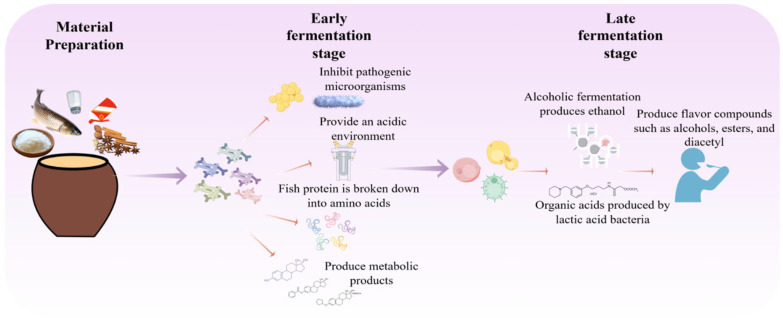
Fermentation Process of Pickled Fish.

Fermented fish is a traditional preserved food of the Dong ethnic group in southwestern China, typically prepared by anaerobically fermenting fresh fish with glutinous rice, chili peppers, Sichuan peppercorns, and seasonings in sealed jars. The product exhibits a distinct sour-umami taste and a complex aroma [90]. Its fermentation is driven by a core consortium of LAB and yeasts, along with other bacteria such as *Staphylococcus* and *Bacillus*. The fermentation process of Dong fermented fish is depicted in Figure 3.

During the initial fermentation stage, lactic acid bacteria (primarily *Lactobacillus plantarum*, *Lactobacillus brevis*, and *Lactobacillus curvatus*, which are commonly found in fermented fish products [36]) proliferate rapidly. They produce large amounts of lactic acid, directly forming the characteristic sour flavor while inhibiting the growth of spoilage and pathogenic bacteria, thereby establishing a biologically selective environment that ensures product safety [91]. Simultaneously, the acidic environment promotes moderate hydrolysis of fish muscle proteins, softening the tissue and improving texture.

As fermentation progresses and the environment becomes highly acidic, acid-tolerant yeasts—including species such as *Saccharomyces cerevisiae*, *Hansenula anomala*, *Pichia* spp., and *Candida* spp.—begin to thrive [92]. These yeasts utilize residual carbohydrates or metabolites to produce ethanol. Consistent with the shared mechanisms summarized in Section 2.4, this ethanol reacts with LAB-generated organic acids to form volatile esters, such as ethyl acetate and ethyl lactate [93]. These ester compounds are largely responsible for the fruity, floral, and wine-like notes that complement the foundational sourness, collectively creating the complex, well-rounded flavor profile typical of high-quality Dong fermented fish.

Recent omics-based studies have helped characterize the metabolic basis of flavor development and its association with microbial succession in fermented fish. Volatilomics and metabolomics analyses suggested that fermentation is accompanied by the accumulation of umami-related amino acids, succinic acid, and peptides, together with changes in taste-active nucleotides and key volatile compounds [94]. In parallel, combined HS-SPME-GC-MS and 16S rRNA sequencing with correlation network analysis indicated that alcohols, nitrogen-containing compounds, aldehydes, and esters are major volatile categories, and that specific bacterial taxa are associated with characteristic aroma compounds [95]. Notably, these omics results mainly provide correlative evidence; therefore, targeted validation remains necessary to confirm functional interactions. Supporting this point, co-culture experiments showed that yeast can promote LAB growth and shorten the lag phase, likely by increasing soluble nitrogen sources (e.g., peptides), while co-metabolism of amino acids (e.g., glutamic acid and aspartic acid) may contribute to volatile formation [96].

### 3.2. Milk Tofu: The “Flavor Shaping” by Lactic Acid Bacteria and Molds

**Figure 4:**
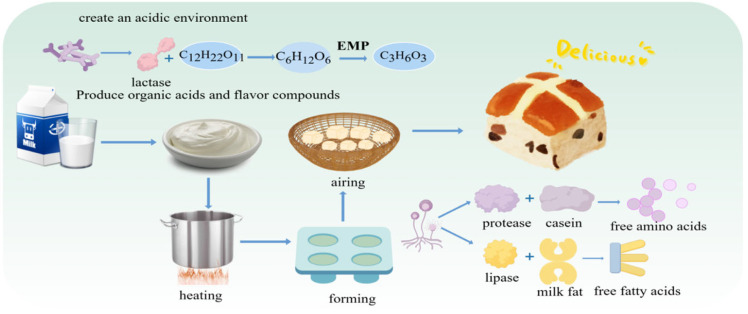
Fermentation Process of Mongolian Milk Curd.

Mongolian milk tofu, known as “Huruda” in Mongolian, is a traditional dairy product made from cow’s, sheep’s, or horse’s milk. It is produced by first fermenting the milk into yogurt via LAB, followed by heating, pressing, molding, and air-drying [97]. This process is not driven by a single microorganism but relies on a symbiotic community comprising LAB, yeasts, and molds. Through a series of synergistic, competitive, and antagonistic interactions, these microbes collectively drive key biochemical transformations, including protein conversion, lipid hydrolysis, flavor compound formation, and pathogen inhibition, ultimately shaping the product’s distinctive texture, flavor profile, and safety characteristics. The fermentation process of milk curd is shown in Figure 4.

Microbial interactions during Huruda production can be broadly described in three stages. Stage 1: Lactic Acid Fermentation. LAB like *L. bulgaricus* and *S. thermophilus* dominate initially. Their rapid lactose metabolism produces large amounts of lactic acid, causing a sharp pH drop. This acidification serves two key purposes: (1) it coagulates milk proteins (e.g., casein) to form the foundational gel structure of the product, and (2) creates a selective acidic environment that inhibits spoilage and pathogenic microorganisms, ensuring biosafety and securing LAB’s ecological dominance [98].

Stage 2: Yeast Fermentation. As pH decreases, acid-tolerant yeasts (e.g., *K. marxianus*, *S. cerevisiae*) become active. Their interaction with LAB is crucial for flavor development. Yeast-produced alcohols esterify with LAB-derived acids (e.g., acetic, butyric acid) to form aromatic esters (e.g., ethyl acetate), imparting fruity and floral notes [12]. Yeast metabolism also generates various trace flavor compounds (aldehydes, ketones, sulfur-containing substances) [44], collectively building the rich, multi-layered flavor profile of high-quality milk curd.

Stage 3: Maturation and mold Activity. During shaping and air-drying, environmental molds (e.g., *Penicillium*, *Aspergillus*) may colonize the product. They secrete proteases and lipases that further break down proteins and lipids, releasing free amino acids, peptides, and fatty acids [99]. This enhances umami and richness while providing substrates for secondary flavor reactions (Maillard reaction, lipid oxidation), leading to more complex and mellow flavors [100]. This mold-driven biotransformation, often combined with specific aging techniques, represents a specialized, advanced stage of microbial succession in the fermentation system [101].

### 3.3. Miao Sour Soup: A Lactobacillus-Dominated “Sour-Aroma Ecosystem”

**Figure 5:**
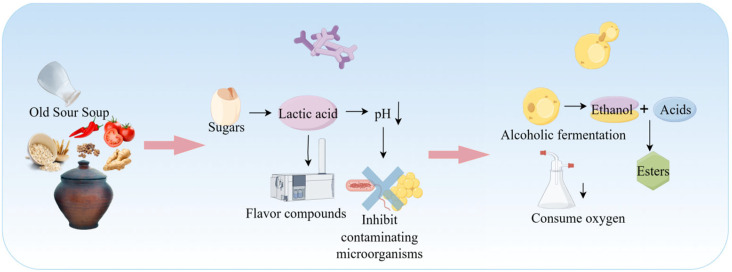
Fermentation Process of Miao Sour Soup.

Miao sour soup is a traditional fermented condiment of southwestern China’s ethnic groups, known for its characteristic sour-umami flavor, digestibility, and extended shelf-life. This is achieved through a locally adapted fermentation system based on plant-based ingredients such as rice, flour, chili peppers, tomatoes, and ginger. The process is driven by a dynamic microbial consortium, predominantly composed of LAB, along with yeasts and minor AAB. Through syntrophic, cross-feeding, and competitive interactions, these microorganisms degrade macromolecules (e.g., starch, protein, cellulose), generating organic acids, free amino acids, volatile compounds, and bioactive metabolites. These products collectively contribute to the product’s reddish/translucent appearance, mellow sourness, and rich, lingering umami taste [102]. The fermentation process of Miao sour soup is shown in Figure 5.

This fermentation system represents a typical microecosystem with LAB as its absolute core. The dominant LAB populations mainly include *Lactobacillus sporogenes*, *Lactobacillus plantarum*, *Lactobacillus acidophilus*, and other Lactobacillus species [103]. During the initial fermentation stage, lactic acid bacteria rapidly metabolize fermentable sugars released from raw materials (such as glucose and maltose) to produce lactic acid through homofermentative or heterofermentative processes. This causes a sharp drop in the pH of the fermentation medium, effectively inhibiting the vast majority of spoilage microorganisms and potential foodborne pathogens while promoting the release of intracellular flavor precursors and nutrients [104]. The metabolic activities of LAB contribute a delicate tartness and a layered, complex sour aroma [105]. Furthermore, the combination of multiple LAB metabolites yields an acidity profile that is not sharp or one-dimensional, but rather mild, well-rounded, and characterized by a sophisticated aromatic bouquet [106].

As the initial sugar content declines and the acidic environment stabilizes, the ecological role of yeasts becomes increasingly prominent. Yeasts metabolize residual sugars and generate ethanol. As described in Section 2.4, this ethanol reacts with LAB-derived organic acids (e.g., lactic and acetic acids) to yield volatile esters such as ethyl acetate and ethyl lactate. These compounds impart characteristic fruity and wine-like aromas, which are key contributors to the ester-driven fragrance of sour soup. In a related study, Zhang et al. [107]. identified 35 volatile organic compounds in fermented sour soup, including 11 alcohols, 9 esters, 6 aldehydes, 5 ketones, 2 acids, 1 furan, and 1 ether, which collectively shape its complex aromatic profile.

Beyond aroma formation, yeast enzymatic activity significantly enhances flavor complexity. Yeast-secreted proteases and lipases degrade proteins and lipids, liberating free amino acids (e.g., glutamic acid, aspartic acid) and free fatty acids. These compounds function both as direct flavor contributors and as essential precursors for subsequent Maillard and Strecker reactions. Moreover, the limited consumption of organic acids and the buffering capacity of yeast metabolites modulate pH within the fermentation system, preventing over-acidification and thus contributing to long-term microbial stability and ecological equilibrium.

### 3.4. Manchurian Kombucha

**Figure 6:**
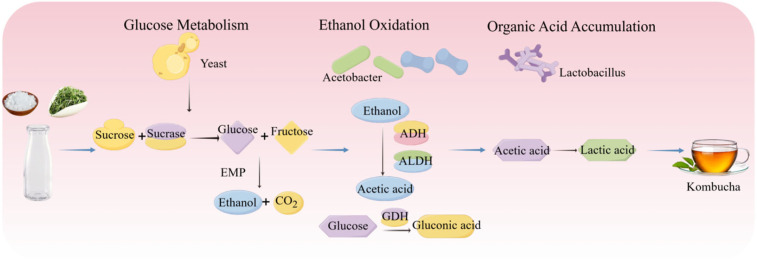
Fermentation Process of Kombucha.

Kombucha is a traditional fermented tea beverage with origins in Northeast China thousands of years ago, and has since gained worldwide popularity [108]. It is produced by fermenting sweetened tea using a symbiotic culture of bacteria and yeast (SCOBY), which generates a range of bioactive compounds and imparts its characteristic flavor profile. This process is driven by the interactions of multi-species microbial communities, primarily comprising yeasts (e.g., *Saccharomyces* spp.), acetic acid bacteria (e.g., *Acetobacter* spp.), and lactic acid bacteria (e.g., *Lactobacillus* spp.) [109]. Key metabolites such as ethanol, acetic acid, gluconic acid, and other organic acids are closely linked to both the distinct sensory properties and the potential health-related functions of kombucha [110]. The fermentation process of kombucha is shown in Figure 6.

During the fermentation of kombucha, yeasts, AAB, and LAB form a dynamic symbiotic network driven by a sequential metabolic cascade of “sugar catabolism–ethanol oxidation–organic acid accumulation.” The interplay of their metabolites and shifting environmental parameters collectively governs the progression of fermentation. In the initial phase, yeasts such as *Saccharomyces cerevisiae* and *Saccharomyces boulardii* hydrolyze sucrose and convert it to ethanol and CO_2_ [111]. Subsequently, consistent with the oxidative mechanisms described in Section 2.4, AAB (e.g., *Acetobacter* spp.) become increasingly active, oxidizing ethanol to acetic acid and directly converting glucose to gluconic acid [112]. In the later stages, LAB such as *Lactobacillus plantarum* further modulate acidity by converting acetic acid into lactic acid, thereby extending the metabolic chain and contributing to the overall acid profile [113]. Moreover, cross-feeding interactions among LAB, yeasts, and AAB significantly enhance the functional resilience and metabolic stability of the fermentation system. Notably, certain LAB strains can metabolize acetic acid to produce lactic acid, which helps regulate environmental acidity and creates favorable conditions for the growth of other community members [114,115]. These relationships are often inferred from compositional or correlative evidence, and targeted validation would strengthen causal interpretation.

The bioactive components in kombucha are derived both from the tea leaves themselves—such as polyphenols, polysaccharides, vitamins, minerals, and amino acids—and from the metabolic activities of the microorganisms involved in fermentation. Key bioactive compounds generated through microbial action include polyphenols, organic acids, vitamins, enzymes, and functional proteins such as bacteriocins [116]. In a study by Jafari et al. [117], black tea fermented at room temperature for 14 days showed increased invertase activity during fermentation, resulting in elevated polyphenol content and enhanced antioxidant capacity. Cardoso et al. [118] reported that kombucha prepared from black tea exhibited stronger antioxidant activity compared to that from green tea. The accumulation of lactic acid significantly lowers the pH of kombucha, thereby enhancing its antimicrobial properties [119]. During fermentation, certain LAB and yeast strains synthesize B-complex vitamins, such as thiamine (vitamin B_1_) [120]. Additionally, the presence of LAB facilitates the production of bioactive peptides and bacteriocins (e.g., nisin), which exhibit broad-spectrum antimicrobial activity against pathogenic and spoilage microorganisms like *Listeria* and *Staphylococcus aureus* [121].

## 4. Conclusions and Outlook

Microbial interactions constitute not only the fundamental biological basis for the development of flavor and quality in traditional fermented foods, particularly those reviewed here from China’s ethnic minorities, but also serve as a critical link between natural ecosystems, ethnic food cultures, and food science. Analysis of representative examples—including Dong sour fish, milk tofu, Miao sour soup, and Manchurian kombucha—demonstrates that diverse microbial groups establish stable yet dynamic fermentation ecosystems through mutualistic, commensal, and competitive interactions. These interactions collectively drive substrate conversion, flavor formation, safety assurance, and functional improvement. This observation supports the view that fermentation is essentially a cooperative metabolic network supported by microbial communities through functional niche partitioning. The stability and functional diversity of this network directly govern the sensory properties, nutritional quality, and technological robustness of fermented food products.

### 4.1. Fermented Foods and Human Health

In food fermentation processes, bacteria, yeasts, and molds play essential roles in decomposing complex substrates and generating bioactive metabolites with significant health benefits. These range from supporting digestive function to modulating immune responses [122]. Moreover, recent research is increasingly focused on exploring novel and less-characterized microbial strains to achieve more specific and targeted health outcomes. Often isolated from distinct ecological niches or identified through advanced microbiological techniques, these strains possess unique metabolic profiles that may confer therapeutic advantages extending beyond general gut health [123]. In contrast to traditional probiotics, which primarily aim to maintain gut microbiome equilibrium [124], these emerging strains are being investigated for their potential in addressing specific health conditions, including mental well-being [125,126], immune regulation [127,128,129], metabolic health [130,131], and the prevention of chronic diseases such as cardiovascular disorders [132,133] and diabetes [134,135]. By elucidating the distinct microbial transformations and bioactive compounds produced by such strains, researchers are paving the way for applications in precision nutrition and personalized health. This progress holds promise for developing targeted, strain-specific fermented foods and supplements tailored to individual health needs, thereby opening new frontiers at the intersection of fermentation science and human health.

### 4.2. Drawbacks in Traditional Fermented Food Production Methods

Traditional food fermentation, while embodying rich culinary heritage, faces limitations in meeting modern technological standards and consumer expectations [136]. A key challenge is the low standardization of production, which leads to inconsistent quality across batches and regions due to reliance on natural conditions and artisanal expertise, hindering large-scale industrialization [137]. Secondly, food safety remains a concern, as open fermentation environments risk contamination by pathogens or toxigenic molds, and inadequate process control may promote the formation of harmful compounds such as biogenic amines. The lack of real-time monitoring and prolonged fermentation cycles further reduce efficiency and increase costs [138]. Additionally, many traditional products depend on high salt or sugar content for preservation, conflicting with contemporary dietary guidelines that emphasize reduced sodium and sugar intake, thereby limiting their alignment with modern health-conscious trends.

### 4.3. Modern Technology Empowers Traditional Fermentation

To address the aforementioned limitations, modern technologies are injecting new vitality into traditional fermentation processes, offering promising pathways for future advancement. In the near term, priorities should focus on improving batch-to-batch consistency, safety control, and standardization; in the longer term, efforts can move toward more designable fermentation systems enabled by microbiome engineering and intelligent process control. Key research and development directions include:

#### 4.3.1. Precise Microbiome Regulation and Engineered Microbial Consortia

Metagenomics and other high-throughput sequencing technologies enable comprehensive profiling of core microbial communities and their functional roles in traditional fermentations. This knowledge allows for the rational design of defined starter cultures by blending functionally characterized core strains—akin to formulating a microbial “prescription”—which can fundamentally enhance fermentation consistency, product stability, and safety [139]. Synthetic microbial ecology further supports the construction of tailored multi-strain consortia that work cooperatively to efficiently produce target flavor compounds or bioactive ingredients [140].

#### 4.3.2. Real-Time Monitoring and Intelligent Process Control

The deployment of advanced sensor systems enables real-time tracking of key fermentation parameters such as pH, temperature, microbial biomass, and specific metabolite concentrations. Coupled with big-data analytics and artificial intelligence (AI), fermentation dynamics can be modeled and predicted, allowing for adaptive optimization and intelligent control of the process. AI-driven systems can autonomously adjust operational parameters to ensure consistent product quality across batches [141].

#### 4.3.3. Targeted Flavor and Health-Oriented Design

Metabolic engineering strategies [142] can be applied to genetically modify or adaptively evolve production strains, enhancing their capacity to synthesize desirable flavor compounds while minimizing off-flavors or harmful metabolites. Additionally, novel fermentation agents [143] can be developed to rapidly establish dominance in low-salt or low-sugar environments, effectively suppressing contaminating microorganisms and enabling the production of fermented foods that align with modern health-conscious dietary preferences.

Looking forward, the production of traditional fermented foods is poised to evolve from an empirically dependent, environmentally variable process into a transparent, predictable, and designable system—termed “intelligent fermentation.” This paradigm shift is rooted in a deeper understanding of microbial ecology and fermentation dynamics, supported by advanced sensing, data integration, and bioprocess control. By integrating modern science with traditional wisdom, intelligent fermentation can retain the unique sensory and cultural attributes of heritage foods while systematically addressing their historical limitations. Ultimately, this approach will provide global consumers with fermented products that are safer, more consistent, and nutritionally tailored, with diverse sensory profiles [144].

## Data Availability

No new data were created or analyzed in this study. Data sharing is not applicable to this article.

## Abbreviations

The following abbreviations are used in this manuscript:

AAB	acetic acid bacteria
LAB	lactic acid bacteria
EMP	Embden–Meyerhof–Parnas
LA	Lactic Acid
ADH	Alcohol Dehydrogenase
ALDH	Aldehyde Dehydrogenase
GDH	Glucose Dehydrogenase
